# COVID-19 and the Elderly: Who Cares?

**DOI:** 10.3389/fpubh.2020.00151

**Published:** 2020-04-21

**Authors:** Florian Fischer, Lea Raiber, Claudia Boscher, Maik H.-J. Winter

**Affiliations:** Institute of Gerontological Health Services and Nursing Research, Ravensburg-Weingarten University of Applied Sciences, Weingarten, Germany

**Keywords:** corona, pandemic, SARS-CoV-2, public health, aging, nursing care

## Introduction

Reports and discussions about the current situation and state of knowledge, information about risks and protective behaviors, and predictions of future scenarios related to COVID-19 are almost omnipresent in the media these days (1). Until now, these discussions have mainly focused on potential overloading of the healthcare system and economic losses, the latter being reinforced by political measures (e.g., lockdowns, curfews, and the closure of non-essential businesses). Although not all the details about its epidemiology are yet clear, deaths related to COVID-19 primarily occur among the elderly and mainly among those with concurrent illnesses such as cardiovascular diseases, respiratory diseases, or diabetes (2, 3). For this reason, more emphasis and adequate solutions to the question “Who cares?” with a focus on the elderly in these times of pandemic is needed. This question can be addressed from multiple perspectives.

## Risk Perception and Solidarity

Firstly, the question relates to the risk perceptions of members of society as a whole. The principle of solidarity, which is employed in social health insurance schemes, also applies to the coronavirus pandemic. Everybody—formal and informal caregivers, relatives, and friends—needs to act in solidarity and responsibly to protect the elderly, who constitute a population at particular risk. In order to do so, we need to overcome the paradox of staying together by keeping apart from each other. Social distancing, isolation, and quarantine are critical for slowing the spread of COVID-19 in the absence of pharmacological approaches for prevention or treatment (4). Although social distancing leads to massive negative impacts on the economy (e.g., due to the closure of businesses that are not system-relevant), the need to protect the elderly—and their human rights—must not be ignored. As risk perceptions might differ, society has to counteract misappraisals such as in a recent case when policymakers rejected the idea of social distancing and frankly demanded that older people “sacrifice” themselves for their country's economy (5).

## Political Responsibility

Secondly, the question relates to political responsibility for the measures that are taken by supranational, national, and local authorities for protecting the elderly. This does not only apply to public health authorities, because concerted action within all relevant policy areas is mandatory. Up to this point, we have witnessed shifting responsibilities and divergent recommendations. For example, several countries in Europe have implemented strict rules, not allowing for a comprehensive response at the supranational level of the European Union (6). In addition, subnational variations (e.g., between states/provinces, counties, or cities) and changes over time are visible. This generates confusion and sows doubt among the public. Therefore, a comprehensible implementation of joint activities and the transparent communication thereof is needed. Although the hope of a coordinated global response is low as countries each tackle their own national crises, the “virtual” G20 emergency summit and the recent common considerations within the European Union are first steps.

## Evidence

Thirdly, all activities targeted at reducing the spread of SARS-CoV-2 should be based on the best available evidence. If no evidence is available, thorough accompanying research needs to be undertaken. Furthermore, all of these activities need to be equitable and inclusive (7). Even non-pharmacological interventions require a (health) impact assessment (8)—taking the perspective of the elderly. There is a need to investigate the (unintended collateral) effects on health, society, and the economy of all measures taken. As already mentioned, the measures for containing the spread of COVID-19 are quite heterogeneous, both between and within countries. Several actions explicitly, or at least implicitly, target the elderly, such as prohibiting visits to nursing and care facilities for the elderly. Nevertheless, one also needs to consider adverse unintended effects. For example, closing schools may increase the exposure of elderly people to carriers of SARS-CoV-2: children spending time together outside school may pose a risk to the elderly when grandparents temporarily look after their grandchildren while parents are at work. In addition, there are potential long-term psychological and social effects that may occur due to loneliness caused by quarantine or lockdowns. Therefore, it is highly relevant to practice social distancing but to avoid social isolation. Adverse effects on the elderly may also occur due to border closures by reducing the labor force of live-in caregivers from abroad. This emphasizes the need for further evidence—at least in the aftermath of this pandemic.

## Protecting Caregivers

Finally, but perhaps even more importantly, measures for protecting caregivers in medicine and nursing are needed. Already in the early stages of this pandemic, we are facing a massive shortage of personal protective equipment. This is not only relevant for medical care (e.g., intensive-care units) but also for nursing care in nursing homes and ambulatory nursing. Public fears of contracting COVID-19 have led to multiple retailers and suppliers running out of respirator masks due to the general public purchasing them. However, in times of shortages of nursing staff, we need to take care of both formal and informal caregivers and provide them with adequate protective equipment. Elderly people in need of care are particularly dependent on family and friends and may also rely on the support of voluntary services and social care (9). If we do not take care of nurses and informal caregivers, the system may collapse, either because caregivers transmit the virus to the elderly or because they are no longer able to provide care due to their own illness or time in quarantine (10). This has dramatic effects, as has been visible in the most recent outbreaks of COVID-19 in several nursing homes, leading to a large number of infections and even deaths.

## Recommendation

COVID-19 needs to be understood as a wake-up call to ensure adequate nursing care for the elderly based on evidence, the requirements of an aging population, responsibility, and social welfare. A strong public health response in the form of urgent and joint action is needed to generate (global) preparedness (11) and to protect this at-risk group. There is no other way to combat COVID-19.

## Author Contributions

FF drafted the manuscript. LR, CB, and MW revised it critically for important intellectual content. All authors read and approved the final version.

## Conflict of Interest

The authors declare that the research was conducted in the absence of any commercial or financial relationships that could be construed as a potential conflict of interest.

## References

[1] Ippolito G Hui DS Ntoumi F Maeurer M Zumla A. Toning down the 2019-nCoV media hype – and restoring hope. Lancet Respir Med. (2020) 8:230–1. 10.1016/S2213-2600(20)30070-9 32146924 PMC7129403

[2] The Novel Coronavirus Pneumonia Emergency Response Epidemiology Team The epidemiological characteristics of an outbreak of 2019 novel coronavirus diseases (COVID-19) – China, 2020. China CDC Wkly. (2020) 2:113–22. Available online at: http://weekly.chinacdc.cn/en/article/id/e53946e2-c6c4-41e9-9a9b-fea8db1a8f51 PMC8392929 34594836

[3] Istituto Superiore di Sanità Sorveglianza Integrata COVID-19 in Italia. (2020). Available online at: https://www.iss.it/documents/20126/0/Infografica_09marzo.pdf/1f62ad0a-e156-cf27–309d-26adcb1b52b4?t=1583782049035 (accessed March 26, 2020).

[4] Wilder-Smith A Freedman DO. Isolation, quarantine, social distancing and community containment: pivotal role for old-style public health measures in the novel coronavirus (2019-nCoV) outbreak. J Travel Med. (2020) 27:taaa020. 10.1093/jtm/taaa020 32052841 PMC7107565

[5] The Guardian The Economy vs. Our Lives? It's a False Choice – and a Deeply Stupid One. (2020). Available online at: https://www.theguardian.com/commentisfree/2020/mar/26/coronavirus-us-economyhealth-lives-trump (accessed April 8, 2020).

[6] Anderson M McKee M Mossialos E. Covid-19 exposes weaknesses in European response to outbreaks. BMJ. (2020) 368:m1075. 10.1136/bmj.m1075 32188590

[7] Berger ZD Evans NG Phelan AL Silverman RD Covid-19: control measured must be equitable and inclusive. BMJ. (2020) 368:m1141 10.1136/bmj.m1141 32198146

[8] Ferguson NM Laydon D Nedjati-Gilani G Imai N Ainslie K Baguelin M Impact of Non-Pharmacological Interventions (NPIs) to Reduce COVID-19 Mortality and Healthcare Demand. London: Imperial College COVID-19 Response Team (2020).

[9] Armitage R Nellums LB. COVID-19 and the consequences of isolating the elderly. Lancet Public Health. (2020). 10.1016/S2468-2667(20)30061-X. [Epub ahead of print]. 32199471 PMC7104160

[10] Alfaraj SH Al-Tawfiq JA Altuwaijri TA Alanazi M Alzahrani N Memish ZA Middle East respiratory syndrome coronavirus transmission among health care workers: implications for infection control. Am J Infect Control. (2018) 46:165–8. 10.1016/j.ajic.2017.08.010 28958446 PMC7115310

[11] Jacobsen KH. Will COVID-19 generate global preparedness? Lancet. (2020) 395:1013–4. 10.1016/S0140-6736(20)30559-6 32199074 PMC7270962

